# New Perspectives of Taxifolin in Neurodegenerative Diseases

**DOI:** 10.2174/1570159X21666230203101107

**Published:** 2023-08-15

**Authors:** Rong Yang, Xinxing Yang, Feng Zhang

**Affiliations:** 1 Key Laboratory of Basic Pharmacology of Ministry of Education and Joint International Research Laboratory of Ethnomedicine of Ministry of Education and Key Laboratory of Basic Pharmacology of Guizhou Province and Laboratory Animal Center, Zunyi Medical University, Zunyi, Guizhou, China;; 2 The Collaborative Innovation Center of Tissue Damage Repair and Regeneration Medicine of Zunyi Medical University, Zunyi, Guizhou, China

**Keywords:** Taxifolin, neurodegenerative disease, neuroinflammation, oxidative stress, neuroprotection, Parkinson’s disease, Alzheimer’s disease

## Abstract

Neurodegenerative diseases, such as Alzheimer’s disease (AD), Parkinson’s disease (PD), cerebral amyloid angiopathy (CAA), and Huntington’s disease (HD) are characterized by cognitive and motor dysfunctions and neurodegeneration. These diseases have become more severe over time and cannot be cured currently. Until now, most treatments for these diseases are only used to relieve the symptoms. Taxifolin (TAX), 3,5,7,3,4-pentahydroxy flavanone, also named dihydroquercetin, is a compound derived primarily from *Douglas fir* and *Larix gemelini*. TAX has been confirmed to exhibit various pharmacological activities, including anti-inflammation, anti-cancer, anti-virus, and regulation of oxidative stress effects. In the central nervous system, TAX has been demonstrated to inhibit Aβ fibril formation, protect neurons and improve cerebral blood flow, cognitive ability, and dyskinesia. At present, TAX is only applied as a health additive in clinical practice. This review aimed to summarize the application of TAX in neurodegenerative diseases and the underlying neuroprotective mechanisms, such as suppressing inflammation, attenuating oxidative stress, preventing Aβ protein formation, maintaining dopamine levels, and thus reducing neuronal loss.

## 1 INTRODUCTION

Neurodegenerative diseases, such as Alzheimer’s disease (AD), Parkinson’s disease (PD), cerebral amyloid angiopathy (CAA), and Huntington’s disease (HD) are characterized by motor symptoms and non-motor symptoms of cognitive dysfunction. The main causes of AD and CAA are amyloid β (Aβ) protein deposition and neuroinflammation. Similar to AD, the main pathological manifestations of PD are α-synuclein aggregation and the formation of Lewy bodies, further resulting in the loss of dopamine (DA) neurons. Studies have confirmed that oxidative stress and neuroinflammation exacerbate DA neuronal damage [1]. Moreover, HD is recognized as motor and cognitive dysfunction irreversibly, and HD has a one-half chance of being passed on to the next generation [2]. Currently, various treatments have been tried to treat neurodegenerative diseases, but they cannot be completely cured due to their complicated pathogenesis [3]. Meanwhile, most drugs used to treat these diseases involveside effects. Thus, opening new alternative avenues for treatment is essential.

Taxifolin (TAX), 3,5,7,3,4-pentahydroxy flavanone, also named dihydroquercetin, is a flavonoid widely found in Pinaceae plants, such as *Larch*, *Douglas fir*, and *Cedrus deodara* [4]. Also, TAX is found in seeds of milk thistle and onion [5, 6]. TAX has been listed as a new food raw material internationally. Meanwhile, TAX has been confirmed to be a health additive in food and drug. Besides, TAX has always been applied clinically for the treatment of cardiovascular and cerebrovascular diseases [7]. In addition, numerous studies have demonstrated the pharmacological activities of TAX, which include anti-cancer [8], anti-inflammatory [9], anti-oxidant [10], anti-viral [11], and anti-bacterial effects [12]. Recently, TAX-mediated neuroprotection has been well studied. For example, TAX inhibited Aβ protein formation and ameliorated cerebral blood flow, further facilitating Aβ clearance in the brain and improving cognitive disorder in the mouse models of CAA and AD, respectively [13]. Moreover, TAX reduced reactive oxygen species (ROS) generation, suppressed inflammation, and protected DA neurons in PD rat model [14, 15]. Otherwise, TAX might confer neuroprotection against HD by its anti-oxidant activity *via* network pharmacology analysis [16]. In this review, we have mainly summarized the effects of TAX on neurodegenerative diseases, including PD, AD, CAA, and HD, and the underlying mechanisms.

## 2 OVERVIEW OF TAX

### 2.1 Physicochemical Property of TAX

**Fig. (1):**
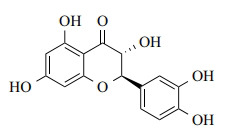
Chemical structure of TAX.

TAX is a flavonoid that was first isolated from *Douglas fir bark* and *Siberian larch* [17]. The stearic structure of the TAX crystal is C_15_H_12_O_7_, with a molecular weight of 304.25 (Fig. **1**). HPLC analysis has revealed that TAX could exist in both trans- and cis-forms, and these trans- and cis-forms crystallize in cells. The melting points of TAX range from 218°C to 253°C [18]. Additionally, TAX is soluble in water-alcohol solutions and polar solvents [19]. Of course, the peculiar structure of TAX provides it with anti-inflammatory and anti-oxidant properties [20].

### 2.2 Pharmacological Actions of TAX

#### 2.2.1 Regulation of Oxidative STRESS

Oxidative stress has been confirmed to be closely involved in the pathogenesis of neurodegenerative diseases. Amounts of physiologic and biochemical stimuli, such as expression of misfolded proteins and perturbation in redox status, could disrupt redox homeostasis and subsequently result in the accumulation of unfolded or misfolded proteins in neurodegenerative diseases. However, the brain response to detect and control oxidative stress was accomplished by a complex network of “longevity assurance processes” integrated into the expressions of genes termed vitagenes. For example, heat shock proteins are a highly conserved system participating in the preservation and repair of correct protein conformation [21]. In addition, the hormetic dose responses were mediated by endogenous cellular defense pathways, such as Nrf2- and sirtuin (SIRT)-related signaling pathways that coordinated adaptive stress responses in the treatment of neurodegenerative diseases [22]. Meanwhile, the emerging role of nitric oxide, hydrogen sulfide gases, and carbon monoxide in hormetic-based neuroprotection against neurodegenerative diseases and their relationship with mitochondrial redox signaling should also be paid more attention [23]. As a flavonoid compound, the carbonyl group in the structure of TAX makes it have an obvious anti-oxidant activity [24]. Previously, TAX was usually used for extending the expiration date of food. Recently, TAX was confirmed to activate Nrf2 signaling to restrain inflammation and oxidative stress in lung injury induced by Benzo[a]pyrene [25]. TAX could prevent chlorpyrifos (CPF)-induced neurotoxicity by up-regulating the level of phosphorylated AMP-activated protein kinase (p-AMPK) and activating Nrf2/heme oxygenase (HO-1) pathway [26]. Moreover, TAX could inhibit ROS production and increase calcium concentration to protect GABAergic neurons [27]. Besides, TAX exhibited apparent inhibition for cell proliferation, ROS overproduction, and NLR family pyrin domain-containing protein 3 (NLRP3) inflammasome activation [28]. Also, TAX produced preventive effects on skin cancer, and the underlying mechanism might be associated with activating Nrf2 signaling *via* an epigenetic pathway. In addition, TAX suppressed cardiac hypertrophy and reduced ventricular fibrosis after pressure overload. These beneficial effects mediated by TAX were at least through the inhibition of ROS production and the activation of extracellular signal-regulated kinase (ERK) 1/2, c-Jun N-terminal kinase (JNK) 1/2, and Smads signaling pathways [29]. On the other hand, TAX improved H_2_O_2_-induced oxidative stress damage and inhibited H9C2 cell pyroptosis [30]. Likewise, TAX reduced the expression of caspase 1, thereby inhibiting the recruitment of macrophages and neutrophils. Beyond that, TAX has been shown to have the ability of decreasing ROS-induced malondialdehyde (MDA) levels, superoxide dismutase and glutathione peroxidase expressions [31]. Another evidence also demonstrated that TAX mitigated acute liver injury induced by CCl_4_ in mice *via* reducing MDA levels and increasing the expression of anti-oxidant enzyme activities [32]. In cisplatin-induced rat pulmonary damage, TAX conferred protection through its anti-oxidative stress actions [33, 34]. Collectively, these findings suggest that TAX generated anti-oxidative stress properties *via* the increased anti-oxidant enzyme activities and the decreased MDA levels.

At present, environmental pollution has become increasingly severe, especially heavy metal pollution. TAX was revealed to produce protection against hexavalent chromium-induced oxidative stress injury and monocyte inflammation *in vitro* [35]. Additionally, TAX was shown to protect retinal pigment epithelium (RPE) cells against oxidative stress-induced apoptosis *via* the activation of Nrf2 signaling and the phase II anti-oxidant enzyme system [36]. It is worth mentioning that TAX generated protection from Di-2-Ethylhexyl Phthalate (DEHP)-induced apoptosis in chicken cardiomyocytes through the anti-oxidative stress effects and regulation of cytochrome P450 (CYP450) expression [37]. Furthermore, TAX prevented acetaminophen (APAP)-induced hepatotoxicity through increasing anti-oxidant enzyme expressions [38].

#### 2.2.2 Anti-inflammation

Flavonoids possess distinct anti-inflammatory properties with beneficial implications for chronic inflammation-induced diseases [39, 40]. Previous studies have confirmed TAX to present remarkable anti-inflammation effects [9]. For example, TAX inhibited LPS-induced immune response in dendritic cells [41]. Moreover, TAX ameliorated caspase-1-induced inflammatory responses in fatty liver cells [9]. In addition, TAX regulated T helper (Th) cells differentiation by inhibiting Notch1 and Jak2/Stat3 pathways activation to treat psoriasis [42]. In the experimental periodontitis of rats, TAX suppressed inflammatory factors’ production to attenuate periodontitis [43]. Similarly, TAX ameliorated iron overload-induced hepatocellular injury by reducing the production of pro-inflammatory cytokines [44]. In various diabetes animal models, TAX was confirmed to regulate postprandial hyperglycemia through its anti-inflammation and anti-oxidant properties [45]. In high-fat diet/streptozotocin-induced diabetic nephropathy (DN) rats, TAX was discerned to inhibit caveolin-1/nuclear factor-κB (NF-κB) signaling activation and further relieve DN [46]. Also, TAX improved alcoholic liver steatosis by inhibiting the activation of NLRP3 inflammasome [47]. Several studies have indicated TAX to attenuate homeostasis of glucose, inhibit the overactivation of the renin-angiotensin-aldosterone system (RAAS), and reduce inflammatory response *via* the inactivation of PI3K/AKT signaling pathway *in vitro* [48]. Additionally, TAX suppressed PI3K/AKT/mTOR and transforming growth factor-β (TGF-β)/Smads pathway activation to exert anti-inflammatory and anti-oxidant effects in order to ameliorate CCl_4_-induced liver fibrosis [49].

#### 2.2.3 Anti-virus

In 2016, as the most active anti-oxidant compound extracted from *Larix gemelinii*, TAX was applied for Coxsackievirus B4 (CVB4) prevention, and it produced an anti-virus effect at the early stages of viral reproduction [50]. Furthermore, TAX has been recognized as a possible inhibitor of SARS-CoV-2 replication [51]. Recently, TAX was confirmed to be an effective natural substance for COVID-19 treatment [11].

#### 2.2.4 Anti-bacteria

In addition to anti-virus action, TAX has been reported to exhibit anti-bacterial properties. TAX combined with dapagliflozin was utilized to cure colistin-induced nephrotoxicity in a rat model [52]. Moreover, TAX enhanced the intestinal barrier and modulated gut microbiota by inhibiting NF-κB signaling activation and further generated protection against dextran sulfate sodium-induced colitis [53]. *Methicillin-resistant staphylococcus aureus* (MRSA) is a common and contagious bacterium with hidden risks. TAX was confirmed to generate protection from MRSA-induced mouse pneumonia and prolong the mouse’s life span [54]. *Helicobacter pylori* (HP), a potent strain of bacteria, could worsen stomach ulcers. TAX attenuated HP-induced gastric ulcers [55].

#### 2.2.5 Anti-apoptosis

TAX was found to reduce cadmium (Cd)-induced cytotoxicity and apoptosis [56]. In addition, TAX promoted the osteogenesis of human bone marrow mesenchymal stem cells (HBMSCs) and further inhibited NF-κB signaling pathway activation [57]. Besides, TAX improved bone formation in alveolar bone successfully in the experimental periodontitis model and decreased bone cell apoptosis [43]. In the myocardial ischemia or reperfusion injury rat model, TAX conferred protection through regulating the mitochondrial apoptosis pathway [58].

#### 2.2.6 Anti-DNA Injury

Surprisingly, TAX was revealed to generate protection against DNA damage [59]. Although TAX did not exhibit DNA toxicity *in vitro*, more clinical experiments are still needed to prove it *in vivo* [60]. These findings suggest that TAX combined with other flavonoids might be a noteworthy anti-cancer medicine potentially [61, 62].

#### 2.2.7 Anti-cancer

An interesting finding reported that TAX could recover various biochemical changes caused by cancer, such as body weight, tumor growth, and various metabolic enzyme activities [63]. Also, TAX had been shown to have the capacity of inhibiting proliferation, migration and invasion of highly aggressive breast cancer cells [8]. In addition, TAX suppressed two enzyme activities of 3β-hydroxysteroid dehydrogenase and 17α-hydroxylase/17 in human with IC_50_ value of about 100 *μ*M. Based on this finding, TAX might be serviceable for treating prostate cancer [64]. Furthermore, TAX reduced the expression of ATP-binding cassette subfamily B member 1 (ABCB1) and the function of P-glycoprotein (P-gp) *via* uncompetitive inhibition of rhodamine 123 and doxorubicin efflux, suggesting that TAX might be useful for multiple drug resistance (MDR) to chemotherapeutic agents in cancer [65]. Similarly, TAX suppressed liver cells' abnormal growth and promoted the apoptosis of liver cancer cells, thus producing anti-cancer effects [66]. For lung cancer, TAX attenuated the stemness of lung cancer cells possibly through the inactivation of PI3K and OCT4 signaling [67]. Moreover, TAX improved dimethylbenzanthracene (DMBA)-induced mammary carcinoma *via* down-regulating the aryl hydrocarbon receptor (AhR) signaling pathway [68]. Besides, in the solar UV-induced skin carcinogenesis mouse model, TAX inhibited epidermal growth factor (EGF)-induced cell transformation to produce protection against UV-induced skin carcinogenesis [69]. In addition, TAX was found to block epithelial-mesenchymal transition (EMT) to prevent migration and invasion of gastric cancer cells and thus inhibit tumor growth. The potential mechanism was associated with suppressing the activation of the AhR/CYP450 1A1 (CYP1A1) signaling pathway [70]. *In vitro* studies, TAX exhibited its preventive potential for colon cancer by enhancing the apoptotic signaling in HCT116 and HT29 colon cancer cells [71]. Together, TAX might be a prodrug or a potential adjuvant for cancer treatment [67].

#### 2.2.8 Anti-hyperglycemia

In the diabetic retinopathy (DR) rat model, TAX reduced retinal ganglion blood vessel damage and decreased oxidative stress and inflammation reactions [72]. Pre-administration with TAX improved the postprandial hyperglycemia in rats and also decreased triglyceride absorption by suppressing pancreatic lipase [73]. Recent studies indicate that TAX derivatives are capable of inhibiting the activity of carbohydrate-hydrolyzing enzymes, decreasing carbohydrate absorption in diabetes [74].

#### 2.2.9 Anti-glycation

Advanced glycation end-products (AGEs) formation is associated with type 2 diabetes. TAX could inhibit AGEs production and show the effect of anti-glycation [75]. Additionally, TAX more efficiently inhibited glycation in the collagen-fructose reaction and the elastin-glyceraldehyde reaction than quercetin and luteolin [76].

#### 2.2.10 Other Biological Activities

TAX inhibited the xanthine oxidase activity in the liver to achieve a strong urine-lowering effect, suggesting that TAX might be a potential substance for treating hyperuricemia [77]. Moreover, TAX uncompetitively suppressed kidney 11β-hydroxysteroid dehydrogenase 2 (11β-HSD2) activity against steroid substrates in human and various animal models [78]. Likewise, TAX inhibited osteoclastogenesis *via* restraining NF-κB signaling activation and decreased bone loss in the mouse model of calvarial osteolysis, which suggested that TAX might be one of the potential treatment means for osteoporosis and rheumatoid arthritis [79]. In addition, TAX suppressed obesity by reducing excessive inflammation [80]. In the high-fat feeding mouse model, TAX presented anti-obesity and gut microbiota-modulating effects [81]. In recent years, TAX has been confirmed to alleviate psoriatic dermatitis in the mouse model and inhibit imiquimod-induced cell over-proliferation [82, 83]. Additionally, TAX suppressed mast cell activation by inhibition of AKT/NF-κB and mitogen-activated protein kinases (MAPKs)/cytoplasmic phospholipase A2 (cPLA_2_) signaling pathway, suggesting that TAX could alleviate mast cell-mediated passive cutaneous anaphylaxis reaction [84]. Increasingly, TAX was able to improve skin viscoelasticity, which was used for the skincare component [85]. Moreover, it was well confirmed that flavonoid-conjugated compounds could enhance the regeneration process and repair hair follicles and sebaceous glands after chemical burns. Further, TAX-conjugated acetaldehyde-based agents were found to be more effective than the wound healing agent Olasol. Therefore, TAX conjugated with carbonyl compounds could be used for burn healing [86]. In addition, in chronic obstructive pulmonary disease (COPD) mouse model and CSE-induced human bronchial epithelial (HBE) cells, TAX conferred beneficial effects *via* attenuating oxidative stress and reducing expressions of iron death-related proteins, such as glutathione peroxidase 4 (GPX4) and solute carrier family 7 member 11 (SLC7A11) [87]. Another evidence showed the interaction of TAX to exist with bovine hemoglobin at physiological pH, revealing TAX to be located in the hemoglobin moiety [88]. Furthermore, TAX could interact with amino acids located in the active site, in which TAX was recognized as the most principal lead molecule and its minimal inhibition concentration was ≤ 12.5 μg/mL [89].

Besides, TAX could attenuate oxidative damage caused by UV injury [69]. Also, TAX showed an anti-aging effect [90], indicating the application of TAX to not only be used for the treatment of certain diseases but also applied as a skin care product. Moreover, TAX could be used in combination with other drugs to enhance the therapeutic effects in clinical practice. For example, glucosamine alendronate (GA) combined with TAX appeared to be more effective than GA treatment alone in maintaining the strength of the femur and bone mineral density (BMD) in the osteoporosis rat model [91]. On the other hand, the combination of TAX and andrographolide enhanced the anti-proliferation and anti-cancer effects of andrographolide [92]. In addition, daily application of TAX (1.5 g/kg) improved the anti-oxidant status and promoted the growth of broiler chicken [93]. Also, TAX was confirmed as a food supplement to improve the immune status of gilthead seabream (*Sparus aurata* L.) and activate immune cells *in vitro* [94]. On the other hand, the purified TAX was proved to confer beneficial effects against biofouling bacteria, algal spore germination, and mollusk foot adhesion, respectively. TAX isolated from *Streptomyces sampsonii* PM33 derived from a mangrove forest was found to be a promising candidate for the development of eco-friendly anti-fouling agents [95]. Collectively, TAX exhibited a variety of pharmacological activities, suggesting that more possibilities of TAX would be applied in future clinical practice.

## 3 TAX AND NEURODEGENERATIVE DISEASES

In CNS, TAX was verified to provide neuroprotection against neurodegeneration *via* reducing inflammatory responses [13] and ROS production [96]. However, the underlying neuroprotective mechanisms warrant further exploration.

### 3.1 TAX and PD

PD is one of the most common neurodegenerative diseases, mainly characterized by DA neuronal loss in the substantia nigra [97]. PD patients present motor symptoms, such as static tremor, bradykinesia, and muscle rigidity, and non-motor symptoms, such as depression, constipation, and hypoxia [98]. PD is more common in the older population, affecting up to 1% of the population over 60 years old, increasing with aging and getting worse [99]. Until now, most drugs have been utilized for PD treatment. However, these drugs have limited efficacy and unignorable side effects [100].

Although the pathogenesis of PD is currently poorly defined, age, neuroinflammation, and oxidative stress are the most important factors in PD progression [101, 102]. The anti-Parkinson potential of silymarin has been proven. Silymarin is a mixture of flavonolignans (silybin, isosilybin, silychristin, and siliandrin) and a flavonoid (TAX), suggesting that TAX might be useful for PD [14]. Moreover, TAX was found to maintain DA levels in the brain and attenuate proteasome inhibitor-induced neurotoxicity [103, 104]. In human neuroblastoma SH-SY5Y cells, TAX decreased ROS production and further attenuated cell apoptosis [105]. In addition, TAX was discerned to reduce the production of peroxide in glial cells *via* inhibiting the deamination of monoamine oxidase (MAO) or scavenging free radicals [106]. Recent studies have indicated TAX to ameliorate oxidative stress and inhibit the expression of pro-inflammatory genes, such as IL-1β and TNF-α, *via* the inactivation of NF-κB signaling, and it ultimately improved rotenone-induced PD rat model [15]. Thus, these findings indicate that TAX-mediated dopamine neuroprotection might be related to suppressing pro-inflammatory factors release and reducing oxidative stress-induced neuronal damage [14, 107].

On the other hand, type 2 diabetes is associated with the risk of PD, which might be due to the production of hyperuricemia [108]. Luckily, TAX has been proven to potentially inhibit hyperuricemia [77]. Besides, another increasing risk factor related to PD is cancer. A clinical case report indicated melanoma patients to have a higher risk of PD [109]. TAX was confirmed to attenuate cellular melanogenesis *via* inhibiting tyrosinase enzymatic activity despite increasing tyrosinase protein levels [110]. Since PD not only includes motor symptoms but also presents non-motor symptoms, such as depression and constipation, TAX was proven to reduce the level of 5-hydroxytryptamine and further produce anti-depression effects [111]. In conclusion, TAX generated neuroprotection against PD directly or indirectly. The underlying neuroprotective mechanisms of TAX on PD are shown in Fig. (**2**).

### 3.2 TAX and AD

AD is characterized by the commonest cause of dementia with cognitive impairment. Most cases occur in the elderly more than 60 years old [112]. The significant pathological feature of AD is the accumulation of Aβ protein misfolding. Of course, the pathogenesis of AD is not just the accumulation of Aβ, but is also related to the immune mechanism in the brain. The misfolding and aggregation of Aβ proteins were found to be linked to pattern recognition receptors on microglia and astroglia, and further produced a congenital immune response. This process was accompanied by a release of various inflammatory mediators, and these mediators were closely associated with the progression and severity of AD [113-115]. In addition, Aβ aggregation, neurofibrillary tangles, and neurodegeneration have been verified to be the predominant inflammatory inducers in AD brain, while activated microglia and astroglia have been reported to exacerbate AD progression [116]. However, neuroinflammation could further accelerate neuronal death in the brain. Previous studies have indicated Aβ_42_ activated cPLA2 and prostaglandin E_2_ (PGE_2_) to damage synapses [117, 118]. In Aβ_42_-induced AD mouse model, TAX promoted the clearance of Aβ_42_ and inhibited the expressions of cPLA2 and pro-inflammatory mediators [119]. Moreover, the computational analysis demonstrated that the covalent admixture formed between the oxidized form of flavonoid (+) -TAX and Aβ could chemically react with Aβ-specific recognition motifs and thus exhibit anti-Aβ aggregation properties [120]. Since Hen egg white lysozyme (HEWL) protein production was recognized to be closely associated with Aβ formation, TAX inhibited HEWL fibril formation and then reduced mature Aβ aggregation in a dose-dependent manner [121,122]. Moreover, microcirculation plays an important role in the brain, and the decreased brain capillary density might be another risk factor for cognitive impairment in AD [123]. In a clinical trial, TAX combined with ascorbic acid for the treatment of atherosclerosis improved cognitive dysfunction and microcirculation [124, 125]. Furthermore, TAX was shown to improve cognitive disorders and protect hippocampal neurons in the mouse model of AD [126]. Also, TAX inhibited Aβ formation and promoted its clearance in the brain, and thus ameliorated brain’s cognitive function [127, 128]. Since β-site amyloid precursor protein cleaving enzyme 1 (BACE1) is known to be a rate-limiting enzyme producing Aβ, TAX has been confirmed to be an effective BACE1 inhibitor [129]. In addition, TAX inhibited pro-inflammatory factors production and then attenuated LPS-induced cognitive dysfunctions. The underlying mechanisms were associated with the promotion of SIRT1 activation and the inhibition of phosphorylated-JAK2/phosphorylated-STAT3-coupled NF-κB-linked BACE1 expression [130]. Moreover, TAX expressed the inhibition of intracerebral Aβ generation by suppressing apolipoprotein E (APOE)-ERK1/ 2-Aβ precursor protein axis, which was responsible for the secretion of Aβ [131, 132]. Besides, TAX generated neuroprotection against cognitive dysfunctions *via* its anti-oxidant and suppression of glutathione (GSH) depletion actions [105]. Likewise, TAX was found to be capable of inhibiting the activity of the neurotransmitter acetylcholinesterase and butyrylcholinesterase from blocking the transmission of these two enzymes in the synaptic cleft and ultimately attenuate neuronal damage [133, 134].

Additionally, CAA is related to the development of lobar intracerebral hemorrhages (ICHs). CAA is pathologically characterized by cerebrovascular Aβ accumulation (deposition of Aβ in the tunica media). Further, studies have confirmed Aβ to exist not only in cortical, leptomeningeal arteries, arterioles and capillaries of most CAA patients but also in the venules of brain in rare cases of CAA [135, 136]. Recently, AD and aging have been confirmed to be other risk factors of CAA [137]. Interestingly, the shared role of Aβ deposition in AD and CAA was arguably the clearest instance of crosstalk between neurodegenerative and cerebrovascular disorders. The pathogenic pathways of AD and CAA intersected at the levels of Aβ generation, its circulation within the interstitial fluid, and perivascular drainage pathways and brain clearance. However, their mechanisms of brain injury and disease presentation diverged [138]. Thus, the treatment for CAA would be effective for AD. TAX was confirmed to promote the breakdown of Aβ and inhibit Aβ assembly and oligomer formation. After TAX treatment, the higher blood Aβ level was discerned, suggesting TAX could promote the clearance of Aβ from the brain to the circulation. Therefore, TAX restored cerebral blood flow (CBF) and cerebrovascular reactivity (CVR), and reduced the accumulation of Aβ and cognitive disorders in the CAA mouse model [139]. In addition, TAX was found to elevate blood Aβ level to maintain cerebrovascular integrity in the CAA mouse model [140]. Furthermore, TAX was verified to facilitate disassembly, block oligomer formation, and increase the clearance of Aβ, and then improve memory impairment in a mouse model of CAA [141]. On the other hand, oxidative stress was reported to aggravate CAA. Inhibition of ROS production might be promising and beneficial for CAA treatment [142]. Recently, microglial activation was observed in CAA-related inflammation. It has been well studied that triggering receptor expressed on myeloid cell 2 (TREM2) is expressed only on microglia in the brain, and TREM2 overexpression would aggravate the neuroinflammation in the brain [143]. TAX could suppress oxidative stress and neuroinflammation, and reduce the overexpression of TREM2 to further confer neuroprotection in the CAA mouse model [131]. Collectively, TAX might open new alternative avenues for AD treatment. The potential mechanism of TAX in AD is shown in Fig. (**3**).

### 3.3 TAX and HD

HD is one of the neurodegenerative diseases with unique clinical presentation, including chorea and dystonia, dyscoordination, cognitive decline, and behavioral disorder. Further, studies have demonstrated mutant huntingtin (m*HTT*) to aggregate mitochondrial and metabolism dysfunctions and inhibit the expression of neurotrophic factors, such as brain-derived neurotrophic factor (BDNF) [144, 145]. Also, m*HTT* could inhibit autophagy to clear misfolded Aβ proteins and further damage neurons. Therefore, at present, the use of genetic techniques to reduce transcription of m*HTT* mRNA is the main therapeutic approach. Additionally, in HD patients and HD mouse models, glial cells’ activation and up-regulation of the mRNA levels of various pro-inflammatory factors were discerned [146]. Thus, inhibition of glial cells’ activation and NF-κB signaling pathway has been considered to be a potential avenue to treat HD [147, 148]. Recent studies have confirmed TAX to be a potentially promising alternative for HD treatment due to its anti-oxidant activity [16]. However, the mechanism underlying TAX-mediated neuroprotection against HD has not been reported till yet.

### 3.4 TAX and Other Neurological Diseases

It is well known that cerebral ischemia reperfusion (I/R) injury is related to the production of free radicals, intracellular calcium overload, excitatory amino acid toxicity, high leukocyte aggregation, inflammatory reactions, and lack of high-energy phosphate compounds. TAX could suppress the expression of inflammatory mediators, such as cyclooxygenase-2 (COX-2) and inducible nitric oxide synthase (iNOS), and inhibit the activation of NF-κB signaling and leukocyte infiltration [149, 150]. Besides, two key counter-receptors, macrophage antigen 1 (Mac-1) and intercellular adhesion molecule 1 (ICAM-1), were found to be closely associated with the limited leukocyte infiltration. TAX was capable of inhibiting these two receptor expressions and then improving cerebral I/R injury [151].

## 4 BIOAVAILABILITY OF TAX

Previous studies have found that TAX bound to glyoxalic acid condensation could become a new compound consisting of two TAX unit dimers, having more effective anti-oxidant effects than TAX [152]. In addition, TAX-chitosan compositions were found to present more apparent anti-hypoxia actions in the process of preventive oral therapy under experimental hypoxia conditions than TAX alone [153]. On the other hand, in order to improve the bioavailability of TAX, γ-cyclodextrin (γ-CD) was employed as an inclusion complex. Further, TAX-γ-CD inclusion complex by emulsion solvent evaporation and freeze drying combination method had demonstrated better bioavailability and anti-oxidant capacity than TAX [154]. Moreover, to achieve better water-soluble biological properties, TAX was contained in the loop of β-cyclodextrin (β-CD), and TAX-β-CD could release TAX slowly and support a low concentration of the free form of TAX for long term, thus making TAX penetrate into the bloodstream [155].

## CONCLUSION

Flavonoids have always been one of the important anti-inflammatory and anti-oxidant compounds. In clinical practice, flavonoids have presented promising beneficial effects in various diseases. As a typical flavonoid compound, TAX not only has significant anti-oxidant and anti-inflammatory activity, but also generates anti-apoptosis, anti-bacterial, and anti-cancer capacity. Besides, TAX was confirmed to exert protective effects against cardiovascular and liver diseases. Recently, studies on TAX in CNS have attracted wide attention. Current studies have indicated TAX to improve cognitive and motor impairment in neurodegenerative diseases, including PD, AD, and HD. The underlying mechanisms have been found to be related to clearing Aβ protein, reducing neuroinflammatory response, increasing DA level, and regulating oxidative stress (Table **1**). Additionally, TAX has been confirmed to present a few side effects in various clinical studies. Importantly, TAX and its metabolites could be detected in rat brain [156]. Thus, TAX might have a good safety profile to be an ideal candidate for neurodegenerative disease treatment. However, current studies are limited to clinical and basic experiments. Collectively, TAX extends our understanding of the neuroprotective properties of TAX in neurodegenerative diseases. Since the etiology and pathogenesis of neurodegenerative diseases are complex, more clinical trials of TAX warrant future investigation.

## Figures and Tables

**Fig. (2):**
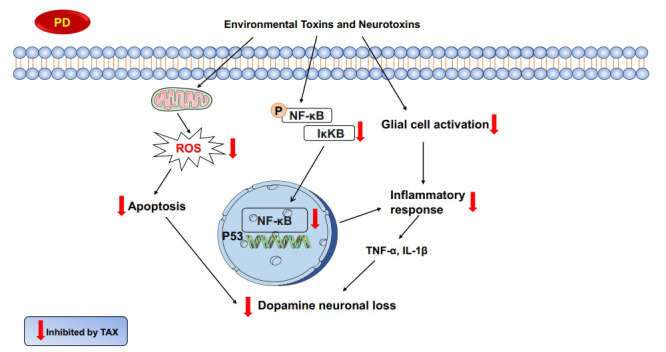
Potential mechanisms underlying TAX-mediated neuroprotection against PD. TAX conferred dopamine neuroprotection *via* decreasing the production of ROS and further attenuating apoptosis, suppressing glial cells-mediated neuroinflammation and inhibiting NF-κB signaling pathway activation.

**Fig. (3):**
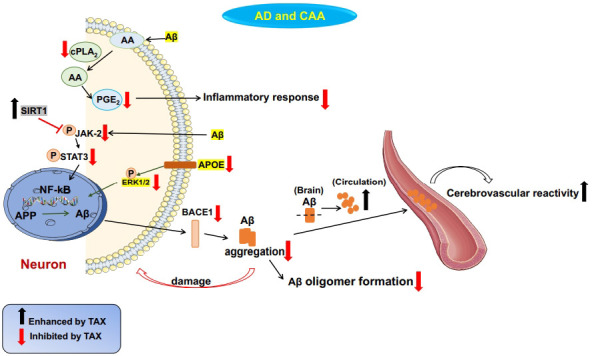
Potential mechanisms underlying TAX-mediated neuroprotection against AD or CAA. In AD, TAX conferred neuroprotection *via* decreasing the level of cPLA2 to reduce the synthesis of PGE_2_, inhibiting neuroinflammation, suppressing the activation of APOE and ERK1/2 signaling to decrease Aβ production, up-regulating SIRT1 expression to inhibit Aβ-induced JAK-2/STAT3 pathway activation, and then decreasing BACE1 expression to ultimately inhibit Aβ aggregation in neurons. In CAA, TAX promoted the breakdown of Aβ, inhibited Aβ oligomer formation, cleared Aβ from the brain to the circulation, and restored the cerebral blood flow (CBF) and cerebrovascular reactivity (CVR).

**Table 1:** Mechanisms underlying TAX-mediated neuroprotection against neurodegenerative diseases.

**Neurodegenerative Disease**	**Biological Target**	**Results**	**References**
Parkinson’s disease (PD)	Rotenone-induced rat model	Inhibiting NF-κB signaling pathway activation and the expressions of inflammatory factors	Akinmoladun *et al.* [15]
	MPTP mouse model	Maintaining DA level	Pérez *et al*. [104]
	SH-SY5Y cell	Decreasing ROS production and further attenuating cell apoptosis	Kim *et al.* [105]
Alzheimer’s disease (AD) and cerebral amyloid angiopathy (CAA)	Aβ_42_-induced SH-SY5Y cell	Reducing cPLA_2_ and PGE_2_ levels	Wang *et al*. [119]
	Aβ_42_-induced mouse model	Inhibiting Aβ_42_ fibril formation	Sato *et al.* [128]
	Neuroblastoma N2a Swe cell line	Downregulating BACE1 expression to inhibit Aβ formation	Park *et al.* [130]
	Mouse primary hippocampal neurons LPS-induced mouse model	Decreasing activation of glial cells and suppressing TREM2 expression Inhibiting the oxidative injury and inflammatory response	Inoue *et al*. [131]
	Tg-SwDI mouse	Reducing Aβ_1-40_ fibril formation and improving cerebrovascular disorders	Saito *et al.* [139]
Huntington’s disease (HD)	Network pharmacology analysis	Anti-oxidant activity	De Oliveira *et al.* [16]

## LIST OF ABBREVIATIONS

AD: Alzheimer’s Disease
Aβ: Amyloid β
BMD: Bone Mineral Density
CAA: Cerebral Amyloid Angiopathy
COPD: Chronic Obstructive Pulmonary Disease
DA: Dopamine
DN: Diabetic Nephropathy
GA: Glucosamine Alendronate
HBE: Human Bronchial Epithelial
HD: Huntington’s Disease
iNOS: Inducible Nitric Oxide Synthase
MDR: Multiple Drug Resistance
MRSA: Methicillin-resistant *Staphylococcus aureus*
PD: Parkinson’s Disease
RAAS: Renin-angiotensin-aldosterone System
ROS: Reactive Oxygen Species
